# Cultured Macrophage Models for the Investigation of Lysosomal Glucocerebrosidase and Gaucher Disease

**DOI:** 10.3390/ijms26062726

**Published:** 2025-03-18

**Authors:** Max Louwerse, Kateryna O. Bila, Martijn J. C. van der Lienden, Arnout Jan M. de Beaufort, Rolf G. Boot, Marta Artola, Marco van Eijk, Johannes M. F. G. Aerts

**Affiliations:** Department of Medical Biochemistry, Leiden Institute of Chemistry, Leiden University, 2333 CC Leiden, The Netherlands

**Keywords:** macrophage, Gaucher disease, glucocerebrosidase, monocyte, lysosome

## Abstract

Macrophages are specialised cells that degrade a range of substrates during their lifetime. In inherited lysosomal storage disorders, particularly the sphingolipidoses, macrophages transform into storage cells and contribute to pathology. An appropriate cultured macrophage model is desired for fundamental research and the assessment of considered therapeutic interventions. We compared commonly used macrophage cell lines, RAW264.7, J774A.1, and THP-1 cells, with human monocyte-derived macrophages (HMDMs) isolated from peripheral blood. Specific lysosomal glucosidases were analysed by enzymatic activity measurements and visualised with fluorescent activity-based probes. Special attention was given to lysosomal glucocerebrosidase (GBA1), the enzyme deficient in Gaucher disease in which lipid-laden macrophages are a hallmark. In macrophage cell lines and HMDMs, various (glyco)sphingolipids relevant to GBA1 activity were determined. Finally, the feasibility of inactivation of GBA1 with a cell-permeable suicide inhibitor was established, as well as the monitoring of uptake of therapeutic recombinant human GBA1. Major differences among various cell lines were noted in terms of morphology, lysosomal enzyme expression, and glycosphingolipid content. HMDMs appear to be the most suitable model for investigations into GBA1 and Gaucher disease. Moreover, they serve as a valuable model for mannose-receptor mediated uptake of therapeutic human GBA1, effectively mimicking enzyme replacement therapy for Gaucher disease.

## 1. Introduction

Lysosomes contain over fifty specialised hydrolases, including exo-glycosidases, which remove external monosaccharides from glycoconjugate substrates [1,2,3,4]. These enzymes are essential in intracellular turnover of endogenous macromolecules such as glycosphingolipids [5,6,7]. Inherited deficiencies in these lysosomal exo-glycosidases are relatively common and result in lysosomal storage disorders (LSDs). One well-studied example is Gaucher disease (GD), one of the most common LSDs, caused by mutations in the GBA1 gene encoding the lysosomal acid β-glucosidase, named glucocerebrosidase (GBA1) [8,9,10]. The clinical manifestation of GD is very heterogeneous. The most common GD phenotype is the non-neuronopathic type 1 variant (GD1) which presents with visceral symptoms such as hepatosplenomegaly, haematological abnormalities and bone involvement with no signs of CNS involvement [11,12,13]. Type 2 (GD2) and type 3 (GD3) GD are characterised by the same visceral symptoms as in GD1 but additionally show neuronopathic abnormalities [13]. GD3, also called the chronic neuronopathic form, has an onset of neurological symptoms in early-adulthood characterised by cognitive impairment, horizontal ophthalmoplegia and generalised tonic seizures [14,15]. GD2, the acute neuronopathic form, has a more extreme and early juvenile onset with severe neurological involvement and patients generally die before the age of 2 years [15,16]. The non-neuronopathic GD can be successfully treated by enzyme replacement therapy (ERT). This therapy involves biweekly infusions of macrophage-targeted recombinant human GBA1 (rhGBA1), resulting in the significant reversal of hepatosplenomegaly and haematological abnormalities [17].

In tissue, macrophages of GD patients may transform into lipid-laden Gaucher cells characterised by lysosomal accumulation of glucosylceramide (GlcCer), the natural substrate of GBA1. These Gaucher cells are the most prominent in spleen, liver and bone marrow of symptomatic GD patients. Accumulating GlcCer is known to be partly deacylated by lysosomal acid ceramidase into glucosylsphingosine (GlcSph), a more water-soluble lyso-sphingolipid that may leave lysosomes and cells, reaching plasma levels that are approximately 200-fold higher in GD patients [18].

Lysosomal glycosidases, including GBA1, operate via the Koshland double displacement mechanism which involves a catalytic nucleophile and acid/base residues [19]. During this process, the glycosidic linkage in the substrate is cleaved, and the sugar intermediate forms a covalent bond with the enzyme’s catalytic nucleophile. Next, the intermediate is hydrolysed (with overall retained stereochemistry) through a nucleophilic attack by water. Based on this reaction mechanism, appropriately configured cyclophellitols and cyclophellitol-aziridines were designed as irreversible covalent (suicide) inhibitors targeting specific retaining glycosidases [20]. The cell-permeable suicide inhibitors are able to impose a GBA1 deficiency on demand in cultured cells when added to the medium [20,21]. When these cyclophellitol-type inhibitors are equipped with a (fluorescent) reporter, they are referred to as activity-based probes (ABPs). These selective fluorescent ABPs allow convenient in vitro and in situ labelling and visualization of various lysosomal glycosidases, including the enzyme GBA1, facilitating detailed analysis of their active forms and localisation [22,23].

Cultured macrophages are a highly relevant research model for investigations on glycosidases involved in sphingolipidoses, in particular GBA1 in GD. Cell lines are often used as models for macrophages including murine RAW264.7 and J774A.1 cells and the human myeloid mononuclear derived THP-1 cells. The THP-1 cells can be differentiated into a more prominent macrophage-like phenotype by incubation with phorbol-12-myristate-13-acetate (PMA) [24,25].

In the present study, we compare human monocyte-derived macrophages (HMDMs), conveniently generated from peripheral blood monocytes by spontaneous differentiation in the presence of human AB serum, with cultured murine RAW264.7, J774A.1 cells and human PMA-differentiated THP-1 cells [26]. Special attention is given to lysosomes and lysosomal glycosidases like GBA1. Lysosomal glycosidases in lysates of various cells were assessed using specific enzyme activity assays with appropriate configured fluorogenic substrates. Furthermore, enzymes were visualised with selective fluorescent ABPs. Specific relevant glycosphingolipids and the deacylated counterparts were assessed by LC-MS/MS. Microscopy was used to compare GBA1 and lysosomes in the various types of macrophages. To induce a GBA1 deficiency, cells were exposed to a specific cyclophellitol-type suicide inhibitor towards GBA1 (inhibitor **1**, Figure S1). Finally, the value of cultured HMDMs was determined by investigation of the uptake and delivery of rhGBA1 to lysosomes.

## 2. Results

### 2.1. Glycosidase Activity Measurements

The HMDMs, PMA-differentiated THP-1 cells, murine RAW264.7 cells and murine J774A.1 cells were cultured as described in Material and Methods. In cell lysates, lysosomal enzyme activities were determined, including α-glucosidase (GAA), β-glucosidase (GBA1), α-galactosidase (GLA), β-galactosylceramidase (GALC), β-glucuronidase (GUSB) and total β-hexosaminidases A and B (HEXA&B). In addition, the activity of GBA2, a non-lysosomal β-glucosidase, was determined [27]. For activity measurements, the corresponding configured fluorogenic 4-methylumbelliferyl-glycoside substrates were used at optimal conditions earlier established for each enzyme. The β-glucosidases GBA1 and GBA2 both hydrolyse 4-MU-β-D-glucose. To distinguish the contribution of the respective enzymes, cell lysates were pre-incubated with the GBA1 specific cyclophellitol-based inhibitor **1** (ME655, Figure S1) [20].

**Figure 1:**
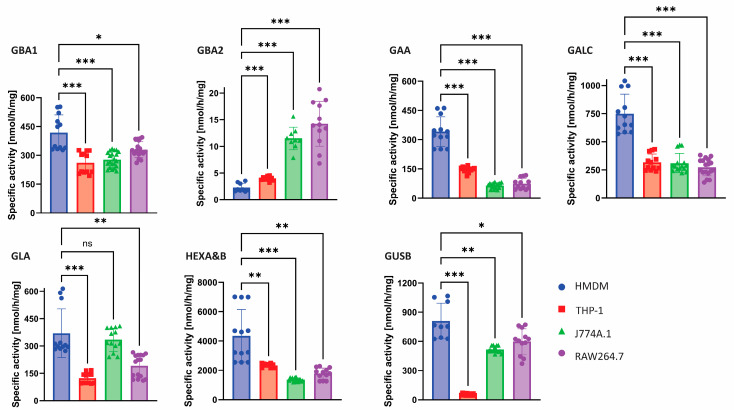
Enzyme activities of a selection of lysosomal enzymes in macrophage models. The activity in lysates of human monocyte-derived macrophages (HMDM), THP-1 macrophages, J774A.1 and RAW264.7 cells was determined by measuring the release of fluorogenic 4-MU from substrates specific for lysosomal/non-lysosomal β-glucosidase (GBA1/GBA2), α-glucosidase (GAA), β-galactosidase (GALC), α-galactosidase (GLA), β-hexosaminidases (HEXA&B) and β-glucuronidase (GUSB). Values are expressed as mean ± SD, at least *n* = 3 with technical triplicates. ns = non significant, * *p* < 0.033, ** *p* < 0.002 and *** *p* < 0.001.

Figure 1 shows that GBA1 activity is highest in HMDM cell lysate. A similar trend is observed for GAA, GALC, HEXA&B and GUSB activities. Strikingly, PMA matured THP-1 macrophages hardly show GUSB activity. Of note, the differentiation of blood monocytes to HMDMs resulted in increased lysosomal enzyme activities (see Figure S2).

### 2.2. ABP Mediated Detection of Glycosidases in Various Macrophages

**Figure 2:**
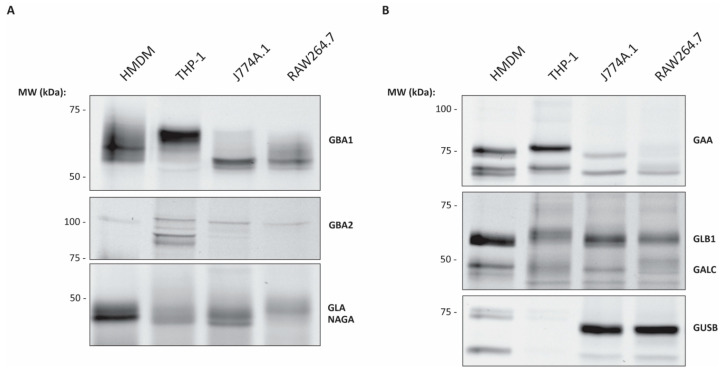
In gel fluorescent detection of a selection of exo-glycosidases labelled with an activity-based probe (ABP) in lysates of human monocyte-derived macrophages (HMDM), THP-1 macrophages, J774A.1 and RAW264.7 cells. (**A**) Labelling with specific ABP **5** for lysosomal β-glucosidase (GBA1), ABP **6** non-lysosomal β-glucosidase (GBA2) and ABP **8** for α-galactosidase A (GLA) and α-*N*-acetylgalactosaminidase (NAGA) (**B**) Labelling with specific ABP **9** for α-glucosidase (GAA), ABP **7** for β-galactosidases (GALC and GLB1) and ABP **10** for β-glucuronidase (GUSB). In the labelling of GAA, GALC/GLB1 and GUSB, before ABP treatment, lysates were pre-incubated with GBA1 inhibitor **1**. Each ABP was incubated for 30–60 min at specific pH for which labelling of the individual glycosidase is optimal at 37 °C. Gels are representative examples of *n* = 3 experiments.

ABPs **5**–**10** (Figure S1) were used to selectively visualise various lysosomal glycosidases (GBA1, GAA, GLA, GALC and GUSB) and GBA2 [22,23]. For this purpose, aliquots of cell lysates were incubated with a specific ABP. After labelling of enzymes, the lysates (equal amount of protein per lane, Figure S3) were subjected to SDS-PAGE gel separation and the fluorescent labelled proteins were visualised by fluorescence scanning. Since the ABPs for GAA, GALC and GUSB upon prolonged incubation also label GBA1, a pre-incubation with the specific non-fluorescent GBA1 covalent inhibitor **1** was performed [20]. When analysing the labelling of GBA1 with ABP **5** (Figure 2A), a striking difference is observed between the labelled enzyme variants in the HMDM and THP-1 cells compared to those in the murine macrophage cell lines. Labelled GBA1 of HMDMs and THP-1 show a more uniform labelling and a higher molecular weight (MW) on SDS-PAGE than their murine counterparts.

The *N*-glycan processing and composition of GBA1 is well known to influence its apparent MW on SDS-PAGE gel [28,29]. We therefore examined whether this is due to a different *N*-glycan composition of GBA1 molecules in the different macrophages. Indeed, differences in molecular weight of GBA1 from various sources disappeared after deglycosylating the enzyme by treatment of cell lysates with PNGaseF (Figure S4). The lower MW band of endogenous GBA1 around 59 kDa visible in the murine macrophage like cells represents the lysosomal form, where *N*-glycans are partially trimmed by lysosomal exoglycosidases [29]. In contrast, GBA1 in THP-1 macrophages exhibits a higher MW form of approximately 68 kDa, indicative of complex sialylated glycan structures present on the enzyme [29].

The amount of labelled GBA2 enzyme with ABP **6** is relatively low in HMDM cells (Figure 2A), which is in line with the enzyme activity analysis. In all cells, GBA2 appears as the earlier reported 110 kDa MW form [30,31]. Additionally, in PMA-matured THP-1 macrophages, two extra lower MW isoforms of around 90 kDa are observed.

The employed β-galactose configured ABP **7** labels both lysosomal β-galactosidases GLB1 (acid β-galactosidase) and GALC (galactocerebrosidase). In all lysates, the mature 64 kDa (lysosomal) form of GLB1 is observed, being most distinct in the HMDMs (Figure 2B) [32]. GALC at 50 kDa is also labelled by the probe. This corresponds to the mature lysosomal form of GALC which is cleaved into a 50 kDa and 30 kDa subunit. Since the active site is present in the 50 kDa subunit, only this form is visualised by ABP [32].

The α-galactose configured ABP **8**, with labels both lysosomal GLA and the lysosomal α-*N*-acetylgalactosaminidase (NAGA) resulting in a band at 49 kDa and 47 kDa, respectively [23]. In Figure 2A the HMDM and J774A.1 cells clearly show two bands just below 50 kDa in which the upper band corresponds to GLA and the lower band to NAGA. The THP-1 and RAW264.7 cells show a much less pronounced separation between the upper and lower band, with THP-1 cells having a seemingly higher labelling of NAGA than GLA while RAW264.7 shows the opposite labelling profile.

The α-glucose configured ABP **9** labels the lysosomal GAA at acid pH, while at neutral pH, the ER α-glucosidase II (GANAB) is labelled [23]. Thus, selective labelling for GAA can be achieved by incubating under acidic conditions. Under these conditions, bands of 76 and 70 kDa are observed, which can be ascribed to lysosomal GAA isoforms (Figure 2B). We were not able to detect the low abundant 110 kDa precursor nor intermediate forms of GAA using our α-glucosidase ABP [23]. The HMDMs show an additional band when compared to the PMA matured THP-1 cells and the murine macrophage-like cells.

Regarding ABP **10** labelling of lysosomal GUSB, HMDMs show two bands (Figure 2B) close to each other which correspond to the 80 kDa full length protein and the 78 kDa C-terminal-truncated isoform [33]. Furthermore, the 64 kDa isoform is also observed in the HMDMs. In THP-1 cells, GUSB labelling is virtually absent, being in line with the enzyme activity analysis (Figure 1). In lysates of murine RAW264.7 and J774A.1 cells, the observed glucuronidase activity deviates from the strong intensity of the ABP labelled protein. Moreover, for these murine cell lines, the lowest isoform of 64 kDa is observed; however, an intense band of around 70 kDa is also seen.

### 2.3. Quantification of Selected Glycosphingolipids in Various Macrophages

Next, we quantified glycosphingolipids and ceramides in the various macrophages. LC-MS/MS was used to quantify LacCer (lactosylcereamide); GlcCer (glucosylceramide); Cer (ceramide); dhCer (dihydroceramide); lyso-LacCer (lyso-lactosylceramide); GlcSph (glucosylsphingosine); Sph (sphingosine) and Spa (sphinganine).

**Figure 3:**
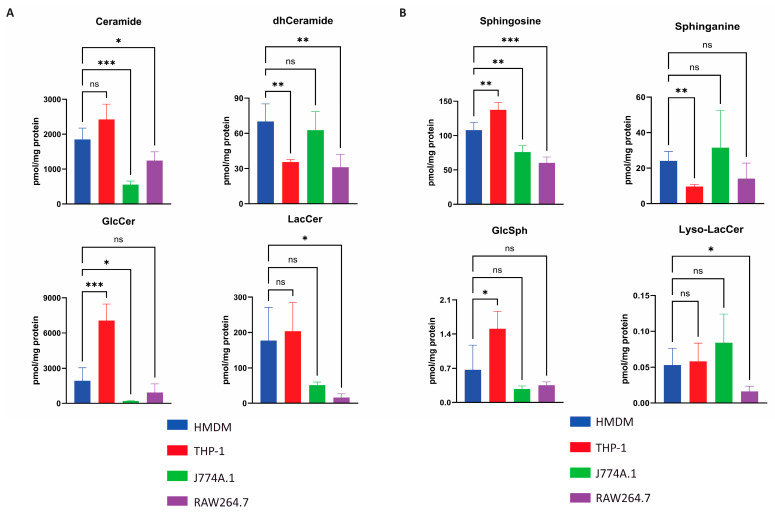
Sphingolipids and lyso-Sphingolipids levels (pmol/mg protein) in macrophage models. Human monocyte-derived macrophages (HMDM), THP-1 macrophages, J774A.1 and RAW264.7 cells (*n* = 3) were harvested, and lipids were extracted and quantified by LC-MS/MS. (**A**) The levels of (glyco)-sphingolipids (**B**) The levels of (glyco)lyso-sphingolipids. Values are expressed as mean ± SD with *n* = 3 and technical duplicates. Statistics were performed if samples were above the limit of detection with significance: ns = non significant, * *p* < 0.033, ** *p* < 0.002 and *** *p* < 0.001.

In case of GlcCer, the highest levels (pmol/mg protein) were noted for THP-1 cells, followed by HMDM cells, and the murine cell lines clearly showed less GlcCer (Figure 3A). For LacCer, a similar trend was observed. The Cer levels were comparable among the various cell types. The product of Cer degradation, sphingosine, was also the lowest in J774A.1 cells (Figure 3B). The dhCer levels were low in all cells, and Spa, an intermediate in de novo dhCer synthesis, was not markedly different among the different macrophages. Lastly, we observed that both GlcSph and lyso-LacCer levels were barely detectable in all of the studied macrophages (Figure 3B). This is expected given that lysosomal function is not perturbed in these cultured macrophages. Small amounts of GalCer and minute quantities of GalSph were detected in HMDM cells (Figure S5).

### 2.4. Glucocerebrosidase Distribution in HMDMs and RAW264.7 Cells

**Figure 4:**
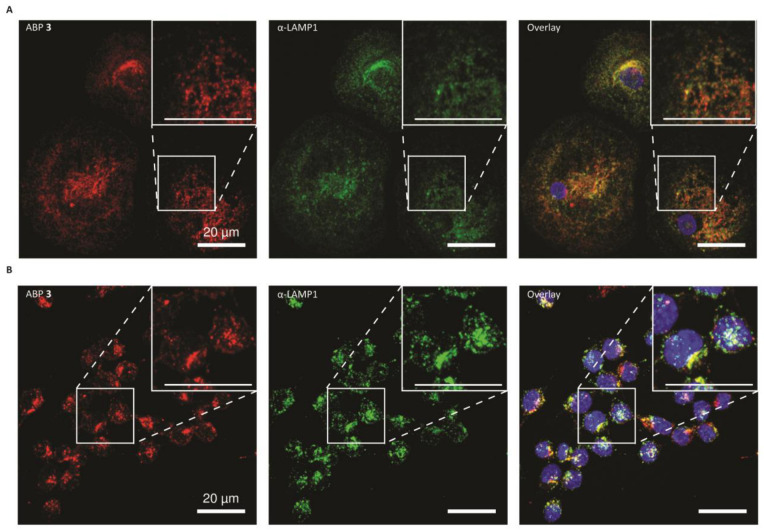
Confocal microscopy of glucocerebrosidase (GBA1) by activity-based probe (ABP) labelling and LAMP1 antibody staining. (**A**) Human monocyte-derived macrophages and (**B**) RAW264.7 cells. Both samples were incubated in situ with 5 nM ABP **3** (Red). Afterwards, samples were fixed, stained with α-LAMP1-antibody (green) and nuclei was stained with 0.5 µM SYTOX Green for (**A**) or 10 µg/mL DAPI for (**B**) (blue). Scale bar: 20 µm.

ABPs can also be used for the in situ labelling of lysosomal enzymes, with the labelling of active GBA1 showing perfect correlation with a GBA1 antibody staining [22]. Therefore, to visualise GBA1 distribution, HMDM and RAW264.7 cells were incubated with ABP **3**. Confocal microscopy revealed that HMDMs have a distinct perinuclear subset of GBA1, while a more peripheral subset can also be observed (Figure 4A). Conversely, the RAW264.7 cells only have a predominant perinuclear localization of GBA1 (Figure 4B). Most notable is the difference of area over which GBA1 is distributed in both cell lines, where the HMDMs have a larger cytosol per cell. Furthermore, in HMDMs, a more tubular network of GBA1 is observed which is absent in RAW264.7 cells.

### 2.5. HMDMs with Inactivated Glucocerebrosidase as Model for Gaucher Disease

**Figure 5:**
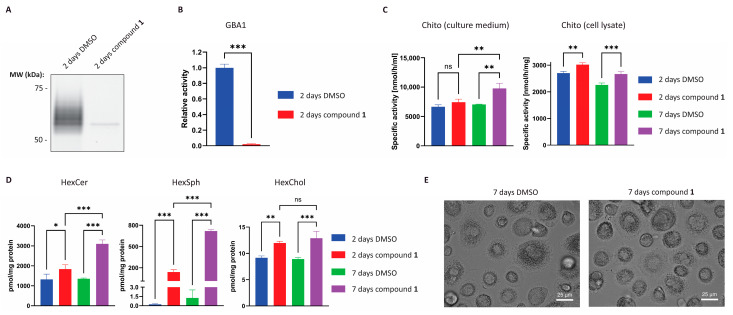
Effect of glucocerebrosidase inhibition by small molecule inhibitor in human monocyte-derived macrophages (HMDMs). HMDMs were cultured in the presence of 100 nM compound **1**, a cell permeable inhibitor of glucocerebrosidase (GBA1), for 2 or 7 days. Fresh medium with inhibitor was added every 2 days and cells were harvest at 21 days post isolation and lysate was used for the analysis of (**A**) unbound GBA1 detection by activity-based probe ABP **5** (**B**) residual relative GBA1 enzyme activity (**C**) chitotriosidase (Chito) enzyme activity in culture medium and cell lysate and (**D**) glycosphingolipids (pmol/mg protein) quantification of hexosylceramide (HexCer), the deacylated for hexosylsphingosine (HexSph) and hexosylcholesterol (HexChol). GBA1 irreversible inhibition with compound **1** for 7 days (**E**) did not show any morphological changes to HMDMs. SDS-Page gel is a representative example of *n* = 3 experiments. Values are expressed as mean ± SD using *n* = 3 for (**B**,**D**), and *n* = 1 for (**C**) all with technical triplicates and ns = non significant, * *p* < 0.033, ** *p* < 0.002 and *** *p* < 0.001. Scale bar: 25 µm for (**E**).

To mimic a GBA1-deficient macrophage (Gaucher cell), the GBA1 selective suicide inhibitor **1** was used to inactivate cellular GBA1 in HMDMs [20]. After 2 days of treatment, GBA1 enzyme activity was undetectable and, in line with this, ABP labelling of GBA1 failed since the catalytic nucleophile of the enzyme was covalently blocked by inhibitor **1** (Figure 5A,B and Figure S6). The faint band observed after GBA1 inhibition could be assigned to newly synthesised GBA1 which is not EndoH resistant (Figure S6). To address functional consequences of GBA1 inhibition, cells were treated with or without inhibitor **1** for 2 days or 7 days and subsequently, the GD biomarker chitotriosidase and lipid composition were analysed. This analysis showed elevated levels of chitotriosidase activity, both in cell lysate and in the culture medium (Figure 5C) [34]. We observed considerable monocyte donor-dependent variation in chitotriosidase production by HMDMs and secretion of the enzyme in the medium. Further experiments with HMDMs from three different donors showed no significant increase in chitotriosidase upon inactivation of GBA1 with compound **1** (see Figure S7). The exposure to inhibitor **1** also caused a time-dependent buildup of HexCer, the endogenous substrate of GBA1 (Figure 5D). The increase in levels of HexSph, the deacylated form of HexCer during GBA1 deficiency, is even more pronounced (Figure 5D). When comparing HMDMs, RAW264.7, J774A.1 and THP-1 cells upon inactivation of GBA1 with suicide inhibitor **1**, all the cells showed a prominent increase in GlcSph and a modest increase in GlcCer levels (Figure S8). More recently, glucosylated cholesterol (GlcChol) has been identified as a new metabolite to be increased in the plasma of GD patients [35]. In line with this observation, inhibition of GBA1 in HMDMs with inhibitor **1** results in a significant increase in HexChol as well (Figure 5D). The observed lipid changes in GBA1 inhibited HMDMs occurred independently of morphological changes (Figure 5E).

### 2.6. Recombinant Glucocerebrosidase Uptake in HMDMs

**Figure 6:**
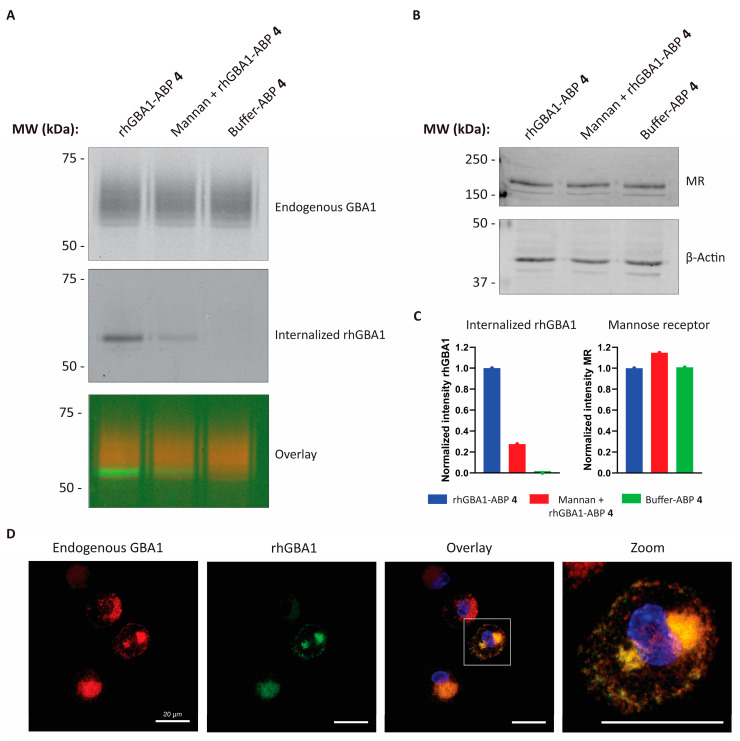
Uptake of therapeutic recombinant human glucocerebrosidase by human monocyte-derived macrophages. Endogenous glucocerebrosidase (GBA1) in human monocyte-derived macrophages (HMDMs) was blocked with ABP **3** and cells were incubated with recombinant human glucocerebrosidase (rhGBA1) pre-labelled with ABP **4** for 6 h. Lysate of HMDMs analysed on SDS-PAGE gel (**A**) showing endogenous GBA1 (red) and internalised rhGBA1 (green) (**B**) western blot of the same gel stained for mannose-receptor (MR) and β-Actin loading control. (**C**) Relative intensities of internalised rhGBA1 and MR normalised towards β-actin of gel and blot seen in (**A**,**B**). (**D**) Confocal microscopy visualization of endogenous GBA1 (red) and internalised rhGBA1 (green) in HMDMs; after fixation, the nuclei were stained with 10 µg/mL DAPI (blue). Scale bar: 20 µm. Gels and Western Blots are representative example of *n* = 3 experiments.

Individuals suffering from GD can, for instance, be treated via a biweekly infusion with human recombinant GBA1 (rhGBA1). The enzyme replacement therapy (ERT) has undergone oligosaccharide remodelling in order to expose terminal mannose residues [36,37]. This modification delivers the recombinant enzyme to macrophages expressing the mannose receptor (MR), also known as CD206. To validate HMDMs as a true model for GD, it is essential to demonstrate their ability to internalize rhGBA1 via the MR. To show this internalization, rhGBA1 was pre-labelled with ABP **4** (green) and endogenous GBA1 was labelled with ABP **3** (red). After the removal of unbound ABP **4**, rhGBA1 was added to HMDMs and incubated for 6 h. Lysates from these HMDMs were subjected to SDS-PAGE separation and fluorescence wet gel slab scanning. The appearance of a band in the BODIPY-green channel indicates that rhGBA1 is able to enter the HMDMs (Figure 6A and Figure S9). When mannan, an endogenous substrate of the MR, is co-incubated with rhGBA1 in the HMDMs, uptake of rhGBA1 is greatly reduced (Figure 6A,C). So, the uptake of rhGBA1 in HMDMs occurs mainly in a MR-dependent fashion. The amount of the MR in HMDMs is not subjected to change when incubated with either rhGBA1, mannan or the buffer control (Figure 6B,C). Lastly, rhGBA1 and endogenous GBA1 were in situ visualised in HMDMs and the signal of both enzymes clearly showed co-localization, indicating that rhGBA1 enters lysosomes in HMDMs (Figure 6D).

## 3. Discussion

Our study compared human monocyte-derived macrophages (HMDMs) and macrophage-like cell lines (PMA differentiated THP-1 macrophages, J774A.1 and RAW264.7 cells) for lysosomal enzyme activities, lysosomal enzyme isoforms and glycosphingolipid composition. The main goal was to establish which kind of cells would serve as the best possible model to study GBA1, including aspects of Gaucher disease (GD). HMDMs with inactivated GBA1 resemble Gaucher cells best, showing, for example, increased expression of chitotriosidase in the case of one monocyte donor. With other donors, chitotriosidase was already high in HMDMs prior to inactivation of GBA1. A macrophage-specific increase in chitotriosidase has not been observed in mouse cell lines. In line with this, HMDMs also best resemble macrophages with a lysosomal apparatus facing lipid pressure. Among conventionally used cell lines, the PMA-differentiated THP-1 macrophages are the most similar to HMDMs. However, there are still some fundamental differences. It is known that the monocyte marker CD14 decreases when human monocytes differentiate to HMDMs while, in THP-1 differentiation, CD14 expression increases [24]. When comparing RAW264.7 cells with HMDMs it becomes evident that HMDMs have a larger cytosol. This makes it easier to identify overlap between GBA1 staining and lysosomal markers such as LAMP1. Again, THP-1 cells are larger in size and thus morphologically the most similar to HMDMs. Intriguing is the noted virtual absence of GUSB in THP-1 cells. Another general disadvantage of cultured cell lines is their genetic instability, resulting in karyotype abnormalities. The cell line RAW264.7 is particularly notorious for acquiring genetic defects, especially at increasing passage number [38]. Non-dividing HMDMs are less likely to show these types of genetic abnormalities.

Some of our findings warrant further discussion. While GBA1 activity levels per mg cellular protein were similar among various analysed cells, the non-lysosomal β-glucosidase (GBA2) activity was the highest among the murine J774A.1 and RAW264.7 cell lines. An explanation for this is presently lacking. It might be related to the species or more likely the different origin and associated phenotype of J774A.1 and RAW264.7 cells compared to HMDMs. The J774A.1. cells stem from an ascitic plasmacytoma in a female BALB/c/mouse, while RAW246.7 cells stem from Mabelson leukemia virus-induced peritoneal lymphoma cells with macrophage-like features from a BAB/14 mouse strain [39,40].

We investigated the value of HMDMs to serve as a cellular model to perform GBA1 centred research. Therefore, we examined GBA1 inactivation in cultured HMDMs by the exposure to suicide inhibitor **1** in the medium (Figure 5). Treated cells showed barely detectable GBA1 activity, as observed earlier for other cell types and zebrafish larvae [20,21]. Additionally, ABP staining of inhibitor treated cells showed a near complete reduction in GBA1 labelling. The inactivation of GBA1 in HDMDs was accompanied by the accumulation of GlcCer and GlcSph, as also observed in GD [5]. Moreover, glycosylated cholesterol is increased in HMDM cells following GBA1-inactivation [5,34]. Finally, we examined the ability of HMDMs to bind and internalize therapeutic rhGBA1. Western blotting and subsequent staining with anti-MR antibody confirmed that HMDMs endogenously express the MR. Also observed was that the uptake of rhGBA1 was competed by mannan (Figure 6A), suggesting the involvement of MR (CD206) as main cellular transporter [41]. Importantly, rhGBA1 was efficiently trafficked to lysosomes and localised alongside endogenous GBA1, demonstrating similar targeting and processing.

In conclusion, our investigation revealed that HMDMs fundamentally differ from macrophage-like cells such as RAW264.7 and J774A.1 cells. In contrast, differentiated THP-1 cells resemble HMDMs the most regarding lysosomal enzyme activity and even morphological characteristics. However, HMDMs with inactivated GBA1 resemble the Gaucher cells encountered in GD patients the best in several aspects (lipids and protein biomarkers). It would be of interest to exploit HMDM cells in future investigations, with or without inactivated GBA1, to reveal mechanisms underlying the pathophysiology of the Gaucher cell and thus GD.

## 4. Materials and Methods

### 4.1. Chemicals

All chemicals used were purchased at Sigma, Schnelldorf, Germany unless stated otherwise. Cerezyme (rhGBA1) was a kind gift by Sanofi-Genzyme. GBA1 inhibitor **1** and **2**, and activity-based probes (ABP) **3**–**10** were synthesised by colleagues at Leiden University (Figure S1) [23].

### 4.2. Cell Culture

RAW264.7 (ATCC, TIB-71) [42] and J774A.1 (ATCC, TIB-67) [43] cells were cultured in Dulbecco’s modified Eagle’s medium (DMEM-High Glucose, Capricorn Scientific, Ebsdorfergrund, Germany) supplemented with 10% foetal calf serum (FCS), 1% (*w*/*v*) GlutaMax (ThermoFisher Scientific, Paisley, Scotland) and 0.2 mg/mL Penicillin/Streptomycin (ThermoFisher Scientific, Paisly, Scotland) at 37 °C under 5% CO_2_.

THP-1 (ATCC, TIB-202) [44] cells were cultured in RPMI-1640 without HEPES (Roswell Park Memorial Institute-1640, Gibco, Paisley, Scotland) supplemented with 10% FCS and 0.2 mg/mL Penicillin/Streptomycin at 37 °C under 5% CO_2_.

Differentiation of THP-1 into macrophages was achieved by seeding cells at 1 × 10^6^ cells/mL in complete medium supplemented with 50 ng/mL phorbol 12-myristate-13-acetate (PMA) for 3 days. Cells were washed once with Dulbecco’s phosphate buffered saline (PBS) and cultured in fresh complete medium for 2 days. Differentiation was verified by morphological changes.

Human monocytes-derived macrophages were cultured in RPMI-1640 without HEPES (Roswell Park Memorial Institute-1640, Gibco, Paisley, Scotland) supplemented with 10% Human Pooled serum (MP Biomedical, Eschwege, Germany) at 37 °C under 5% CO_2_.

### 4.3. Cell Lysis

Cells were cultured as described above until 80–90% confluency. Cells were washed with PBS before harvesting. All the adhering macrophage cells were scraped in PBS and cell pellet was obtained by centrifuging samples 7000× *g* for 10 min. Pellets were stored in −80 °C until further use. Pellets were dissolved in 25 mM potassium phosphate buffer (KPi-buffer, K_2_HPO_4_-KH_2_PO_4_ pH 6.5, supplemented with 0.1% (*v*/*v*) Triton X-100). Cells were lysed by sonication with a Polytron PT 1300D sonicator (Kinematica, Luzern, Switzerland) at 20% amplitude, 1 s pulse and 15 s rest for 5 cycles on ice. Lastly, the protein content of samples was determined with Pierce BCA protein assay kit (ThermoFisher Scientific, Landsmeer, The Netherlands) according to manufacturer’s instructions and absorbance was measured using an Emax Plus microplate reader (Molecular Devices, München, Germany).

### 4.4. Primary Monocyte Isolation

Fresh buffy coats (Sanquin, Amsterdam, The Netherlands) were diluted 2.4× in cell culture grade 0.1% bovine serum albumin in Dulbecco’s phosphate buffered saline (BSA-PBS). Diluted buffy coat was layered on Lymphoprep (Stemcell Technologies, Saint Egreve, France) and centrifugated at 1000× *g* for 15 min. The PBMC-layer was washed with an excess of 0.1% BSA-PBS and centrifuged 750× *g* for 10 min, washed a second time with an excess of 0.1% BSA-PBS and centrifuged 500× *g* for 5 min, followed by a final wash with an excess of 0.1% BSA-PBS to deplete thrombocytes and centrifuged at 250× *g* for 10 min [45]. A 100% stock isotonic Percoll (SIP)-solution was made: 0.9 parts Percoll and 0.1 parts PBS* (289 mM NaCl, 3.60 mM Na_2_HPO_4_ ∙2H_2_O, 76.0 mM KH_2_PO_4_). All further dilutions of 100% SIP were made in Iscove’s Modified Dulbecco’s Medium (IMDM, Capricorn Scientific, Ebsdorfergrund, Germany) + 1% FCS. A density gradient was made with 60% SIP containing the PBMCs as the bottom layer, 45% SIP on top and finally layering 34% SIP in the ratio 1:2:0.8, respectively. Samples were centrifuged at 1750× *g* for 45 min with slow acceleration of the centrifuge and without braking during deceleration in order to not disturb the SIP-layers. After this density centrifugation, the Percoll low density cell fraction (monocyte layer) was collected and washed 3× with 0.1% BSA-PBS. Monocytes in PBS were counted using the TC-20 cell counting system (Bio-Rad, Veenendaal, The Netherlands) according to manufacturer’s protocol. Afterwards, monocytes were seeded in RPMI-1640 (#21875, Gibco, Paisley, Scotland) supplemented with 10% Human Pooled serum (MP Biomedical, Eschwege, Germany) in appropriate plates and cell densities depending on experimental objective. During the first 6 days post isolation, the fresh medium was added on top every 2–3 days. On day 6, the medium was aspirated, samples were washed 3× with 0.1% BSA-PBS and the fresh medium was added every 2–3 days until day 20 when HMDMs were harvested by scraping in PBS.

### 4.5. Activity-Based Probe Analysis

Cells were harvested and lysed as described above. Equal amounts of protein were diluted in 300 mM McIlvaine Buffer (Citric Acid/Na_2_HPO_4_) at optimal pH for the given enzyme in a volume of 20–50 µL and samples were incubated with an excess of ABP **3**–**10** (all probes are equipped with a Cy5 fluorophore) at 37 °C for 30–60 min. Afterwards, 4× Laemmli sample buffer (250 mM Tris-HCl pH 6.8, 16% (*v*/*v*) Glycerol, 20% (*w*/*v*) SDS and 0.02% (*w*/*v*) bromophenol blue and freshly added 10% (*v*/*v*) 2-mercaptoethanol) was diluted to 1× sample buffer in sample and samples were denatured by heating samples 5 min at 98 °C and separated on 7.5–12% SDS-PAGE gels depending on the molecular weight of the enzyme of interest. Wet slab gels were scanned for fluorescence on a Typhoon FLA9500 (GE Healthcare, Eindhoven, The Netherlands) at λ_EX_ = 473 nm and λ_EM_ ≥ 510 nm for BODIPY-green fluorescence; λ_EX_ = 532 nm and λ_EM_ ≥ 575 nm for the BODIPY-red fluorescence and λ_EX_ = 635 nm and λ_EM_ ≥ 665 nm for Cy5 fluorescence. Afterwards, the wet slab gel was stained using Coomassie brilliant blue G250 or R250 for total protein staining and imaged with a ChemiDoc MP (Bio-Rad, Veenendaal, The Netherlands). Data were analysed using Fiji ImageJ version 1.54p.

### 4.6. Western Blotting

Samples were run on SDS-PAGE gels as described above. After wet slab gels were scanned, gels were transferred onto 0.2 µm nitrocellulose membranes (Bio-Rad, Veenendaal, The Netherlands) using Trans-Blot Turbo Transfer System (Bio-Rad, Veenendaal, The Netherlands) at 1.3 A for 10 min. Blots were blocked in 5% BSA-PBS for 30 min at RT. For primary antibody rabbit-α-mannose receptor 1:10,000 (Abcam, Cambridge, United Kingdom, #ab64693), rabbit-α-βActin 1:1000 (Cell Signaling, Leiden, The Netherlands, #4967) or mouse-α-GBA1 1:300 (8E4, made in Aerts-lab [46]) diluted in 5% BSA-PBST were incubated for 18 h at 4 °C. The secondary staining was achieved by incubation for 2 h at RT of donkey-α-rabbit-Alexa647 or donkey-α-mouse-Alexa488 (Invitrogen, Bleiswijk, The Netherlands) 1:10,000 diluted in 5% BSA-PBST. After washing 3× with PBST, blots were imaged with a ChemiDoc MP (Bio-Rad, Veenendaal, The Netherlands) and data were analysed in Fiji ImageJ version 1.54p.

### 4.7. Recombinant Human Glucocerebrosidase Uptake

HMDMs were cultured in 12-well plates at a density of 1 × 10^6^ cells/well as described above. Endogenous GBA1 was blocked by incubating with 100 nM of ABP **3** (BODIPY-red) in cell culture medium for 16 h at 37 °C under 5% CO_2_. Subsequently, 1 µg/µL rhGBA1 (Cerezyme) was diluted in 150 mM McIlvaine pH 5.2 supplemented with 0.2% (*w*/*v*) BSA, 0.1% (*w*/*v*) NaTc and 0.1% (*v*/*v*) Triton X-100 was pre-labelled with 2 µM of ABP **4** (BODIPY-green) for 16 h at 37 °C. Afterwards, unbound ABP **4** was removed by using protein desalting spin columns (ThermoFisher Scientific, Landsmeer, The Netherlands), which, according to manufacturers specifications, can recover proteins >7 kDa or free ABP **4**. The medium was removed from HMDMs and replaced with the medium supplemented with either 100 nM ABP **3** alone or 3 mg/mL Mannan, to block the mannose receptor, and 100 nM ABP **3.** Afterwards, 35 µg of the labelled Cerezyme was added to the cells, or 35 µL of the filtered buffer was added as a control and cells were incubated for 6 h at 37 °C under 5% CO_2_. Samples were harvested, lysed and loaded on SDS-PAGE gel, as described above.

### 4.8. Enzyme Activity Assay

Cultured cells were lysed in 25 mM KPi-buffer pH 6.5 + 0.1% Triton X-100, except for the measurement of GBA2 activity, which required lysis in 25 mM KPi-buffer pH 6.5 without any additives. Cell lysates were sonicated, and protein content was determined as described before. Enzyme activity was analysed in a black flat bottom 96-well plate (Greiner Bio-One, Alphen aan den Rijn, The Netherlands). 25 µL of lysate was mixed with 100 µL 4-MU mix (specific for each enzyme all supplemented with 0.2% *v*/*v* DMSO) in 150 mM McIlvaine buffer (Citric Acid/Na_2_HPO_4_) at pH optimum for each enzyme activity and incubated at indicated times at 37 °C. Substrate mixtures were as follows: Assay GBA1: 3.75 mM 4-MU-β-D-glucopyranoside supplemented with 0.1% (*w*/*v*) BSA, 0.1% (*v*/*v*) Triton X-100 and 0.2% (*v*/*v*) sodium taurocholate in 150 mM McIlvaine pH5.2. Assay GBA2: 3.75 mM 4-MU-β-D-glucopyranoside supplemented with 0.1% (*w*/*v*) BSA in 150 mM McIlvaine pH5.8. Assay α- and β-hexosaminidase: 2.5 mM 4-MU-*N*-acetyl-β-D-glucosamine supplemented with 0.1% (*w*/*v*) BSA in 150 mM McIlvaine pH 4.4. Assay GAA: 3 mM 4-MU-α-D-glucopyranoside in McIlvaine pH 4.5. Assay GLA: 3.75 mM 4-MU-α-D-galactopyranoside supplemented with 0.1% (*w*/*v*) BSA and 250 mM *N*-acetyl-D-galactosamine (BLDPharm, Reinbek, Germany) in 150 mM McIlvaine pH 4.5. Assay GALC: 1 mM 4-MU-β-D-galactopyranoside supplemented with 0.2 M NaCl and 11 µM AgNO_3_ (competitive inhibitor of β-galactosidase [47]) in 150 mM McIlvaine pH 4.5, GUSB; 2.5 mM 4-MU-β-D-Glucuronide in 150 mM McIlvaine pH 5.0. All 4-MU substrates were purchased at Glycosynth (Cheshire, United Kingdom). The following incubation reactions were quenched by the addition of 200 µL 1M glycine-NaOH pH 10.3 and 4-MU emitted fluorescence was measured at λ_ex_ = 366 nm, λ_em_ = 445 nm on a fluorescence spectrometer (LS55, PerkinElmer, Drachten, The Netherlands).

### 4.9. Confocal Microscopy

Cells were grown in 12-well plates (Sarstedt, Breda, The Netherlands) containing a glass coverslip (15 mm diameter) until 70% confluence was reached. Cells were incubated with 5 nM of ABP 8 diluted in medium (specific for each cell line as specified before) for 5 h at 37 °C under 5% CO_2_. Unbound ABP was washed away by washing 3× with PBS and samples were fixated by incubation with 4% Paraformaldehyde (ThermoFisher Scientific, Landsmeer, The Netherlands) in PBS for 15 min at RT. If samples only contained ABP staining, they were immediately incubated with 0.1 mg/mL DAPI for 30 min at RT. After washing in PBS, samples were mounted on a glass microscopy slide (Corning, Amsterdam, The Netherlands) using ProLong Diamond (Invitrogen, Bleiswijk, The Netherlands). If ABP stain was combined with immunocytochemistry, the samples were permeabilised by incubation of 0.1% Tween-20 in tris-buffered saline (TBS) for 30 min at RT. Blocking of samples was performed by incubating in 3% bovine serum albumin in PBS for 30 min at RT. For labelling of LAMP-1 an α-LAMP1 antibody (ab24170, Abcam) 1:500 diluted in 3% BSA-PBS + 0.1% Tween-20 (BSA-PBST) was incubated for 18 h at 4 °C in a humidified environment. For secondary labelling, all antibodies were diluted in 3% BSA-PBST supplemented with 0.1 mg/mL DAPI or 0.5 µM SYTOX Green (Invitrogen, Bleiswijk, The Netherlands); donkey-α-rabbit Alexa488 (Invitrogen, Bleiswijk, The Netherlands) 1:1000 diluted or donkey-α-rabbit Alexa647 1:400 diluted (Invitrogen, Bleiswijk, The Netherlands) were incubated for 1h at RT. After washing in PBS, samples were mounted on a glass microscopy slide using ProLong Diamond and imaged on Nikon Eclipse Ti2 confocal microscope with a 100×/1.49 Numerical Aperture SR HP Apo TIRF oil immersion objective equipped with PMTs detector.

### 4.10. Glycosphingolipid Analysis

Cells were grown in tissue-culture dishes (100 mm diameter, Greiner Bio One, Alphen aan den Rijn, The Netherlands) or tissue culture flask (75 cm^2^, Greiner Bio One, Alphen aan den Rijn, The Netherlands) and washed with PBS and pelleted as described before. Lysates of pellets were prepared in 150 µL LC-MS grade H_2_O (Biosolve, Valkenswaard, The Netherlands). Cells grown in 6-well plates (Greiner Bio One, Alphen aan den Rijn, The Netherlands) were harvested by the addition of 150 µL MS-grade H_2_O to the plate and scraping. 100 µL of lysate was used for lipid extraction while the remaining 50 µL was used to determine protein concentration, as described above. Each sample for lipid extraction was split into 3 separate samples for a technical triplicate. Lipids were extracted and measured using LC-MS/MS as previously described [48].

For glucose and galactose separation, samples were harvested, as described above, and split in technical duplicates. In order to separate glucose and galactose, hydrophilic interaction liquid chromatography (HILIC) separation was employed. Samples were extracted as described previously; however, lipids were resuspended in acetonitrile/methanol (9:1, *v*/*v*) prior to transfer to LC-MS vials [48].

### 4.11. Statistics

All values are represented as mean ± SD. Statistical significance was analysed with Welch one-way ANOVA test with criterion for statistical significance set on *p* < 0.033, unless stated otherwise. Statistics were performed in GraphPad Prism 10.

## Data Availability

The original contributions presented in this study are included in the article/Supplementary Material. Further inquiries can be directed to the corresponding author.

## Supplementary Materials

The following supporting information can be downloaded at: https://www.mdpi.com/article/10.3390/ijms26062726/s1.

## Abbreviations

The following abbreviations are used in this manuscript:

LSDs	Lysosomal storage disorders
GD	Gaucher disease
GD1	Non-neuropathic Gaucher disease type 1
ERT	Enzyme replacement therapy
rhGBA1	Recombinant human glucocerebrosidase
GlcCer	Glucosylceramide
GlcSph	Glucosylsphingosine
ABP	Activity-based probe
PMA	Phorbol-12-myristate-13-acetate
HMDMs	Human monocyte-derived macrophages
GAA	α-glucosidase
GBA1	Glucocerebrosidase
GBA2	Non-lysosomal β-glucosidase
GLA	α-galactosidase
GALC	β-galactosylceramidase
GUSB	β-glucuronidase
HEXA&B	β-hexosaminidases
GLB1	Acid β-galactosidase
NAGA	α-*N*-acetylgalactosaminidase
GANAB	ER α-glucosidase II
MW	Molecular weight
LAMP1	Lysosomal associated membrane protein 1
Cer	Ceramide
dhCer	Dihydroceramide
LacCer	Lactosylceramide
Lyso-LacCer	Lyso-Lactocylceramide
HexSph	Hexosylsphingosine
HexCer	Hexosylceramide
Sph	Sphingosine
Spa	Sphinganine
GlcChol	Glucosylated cholesterol
HexChol	Hexosylcholesterol
MR	Mannose receptor
