# Supplementary material for: Cultured Macrophage Models for the Investigation of Lysosomal Glucocerebrosidase and Gaucher Disease
Source: Int J Mol Sci. 2025 Mar 18;26(6):2726. doi: 10.3390/ijms26062726 (PMC11943430; doi:10.3390/ijms26062726)
Supplement: Supplementary file 1 [file ijms-26-02726-s001.zip › ijms-3474672-supplementary.pdf]

Supplemental information – Cultured macrophage models for the investigation of lysosomal glucocerebrosidase and Gaucher disease.

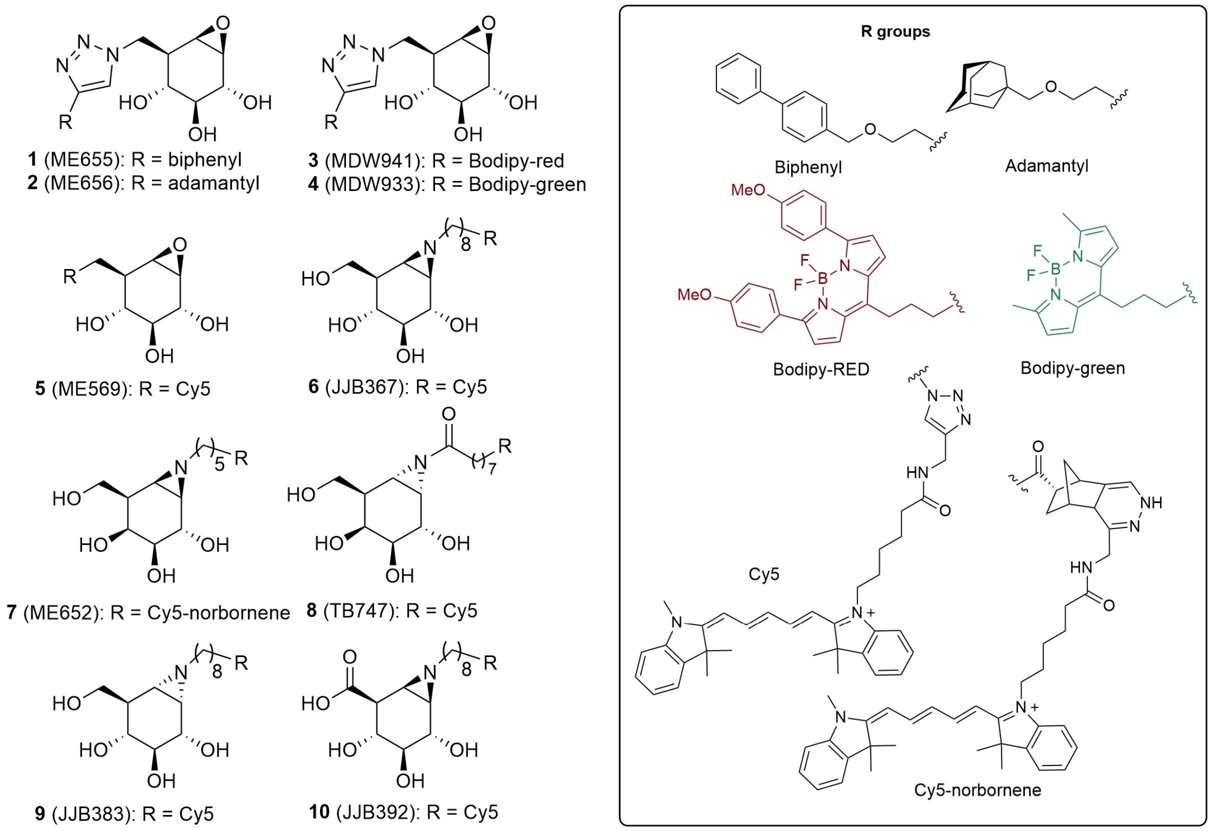

Figure S1. Structures of inhibitors and activity based probes used in this study.

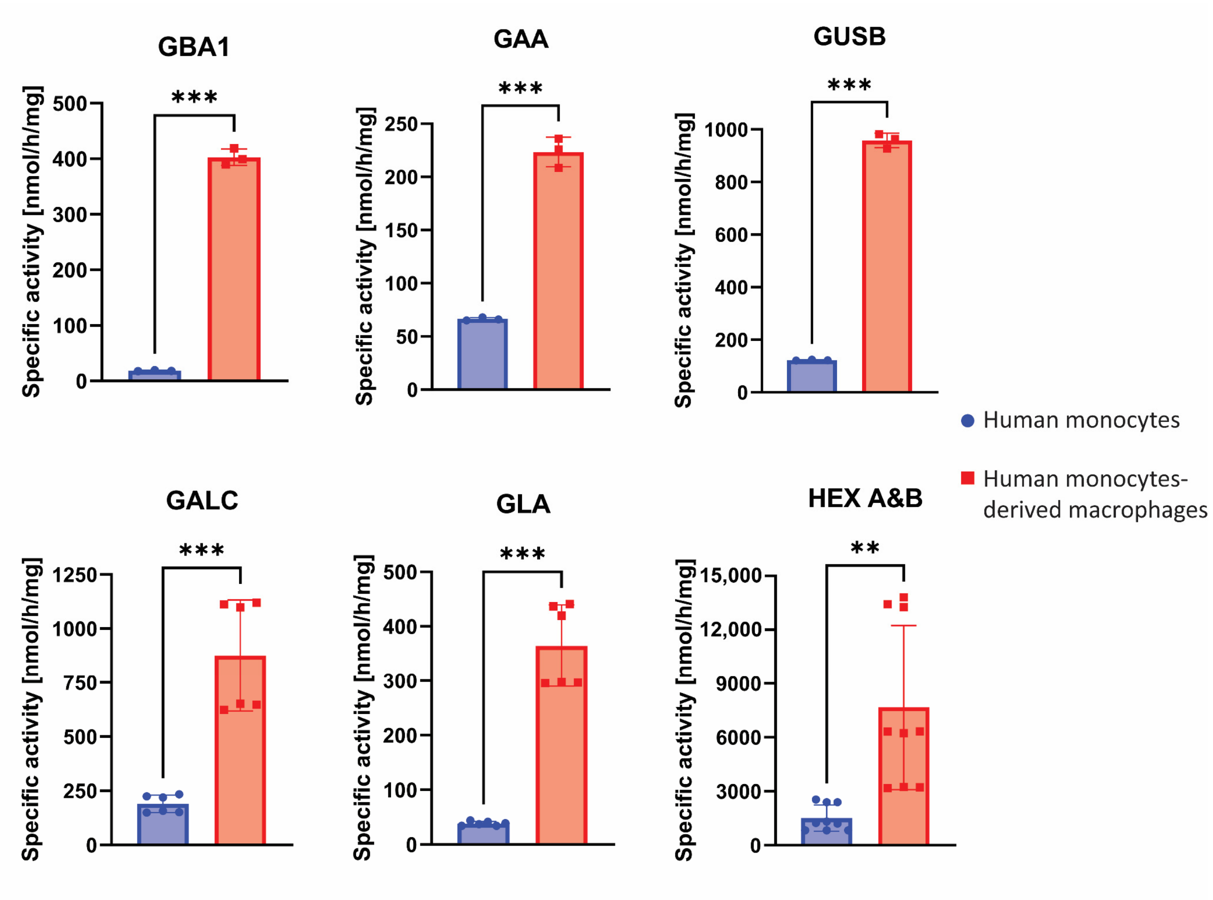

Figure S2. Enzyme activities of a selection of lysosomal enzymes in human monocytes and human monocyte-derived macrophages. The activity of lysosomal enzymes in lysates of human monocytes and the corresponding human monocyte-derived macrophages which have been differentiated for 20-23 days. Enzyme activity was determined by measuring the release of fluorogenic 4-MU from substrates specific for β-glucosidase (GBA1), *α*-glucosidase (GAA), β-glucuronidase (GUSB), *α* & β-hexosaminidases (HEXA&B) β-galactosidase (GALC) and *α*-galactosidase (GLA). Values are represented as mean ± SD with n=1 for GBA1,GAA&GUSB, n=2 for GALC&GLA and n = 3 for HEX A&B with all technical triplicates. For statistical analysis a two-tailed unpaired student's t-test was performed with statistical significance of: $^{*}P < 0.033$, $^{**}P < 0.002$ and $^{***}P < 0.001$.

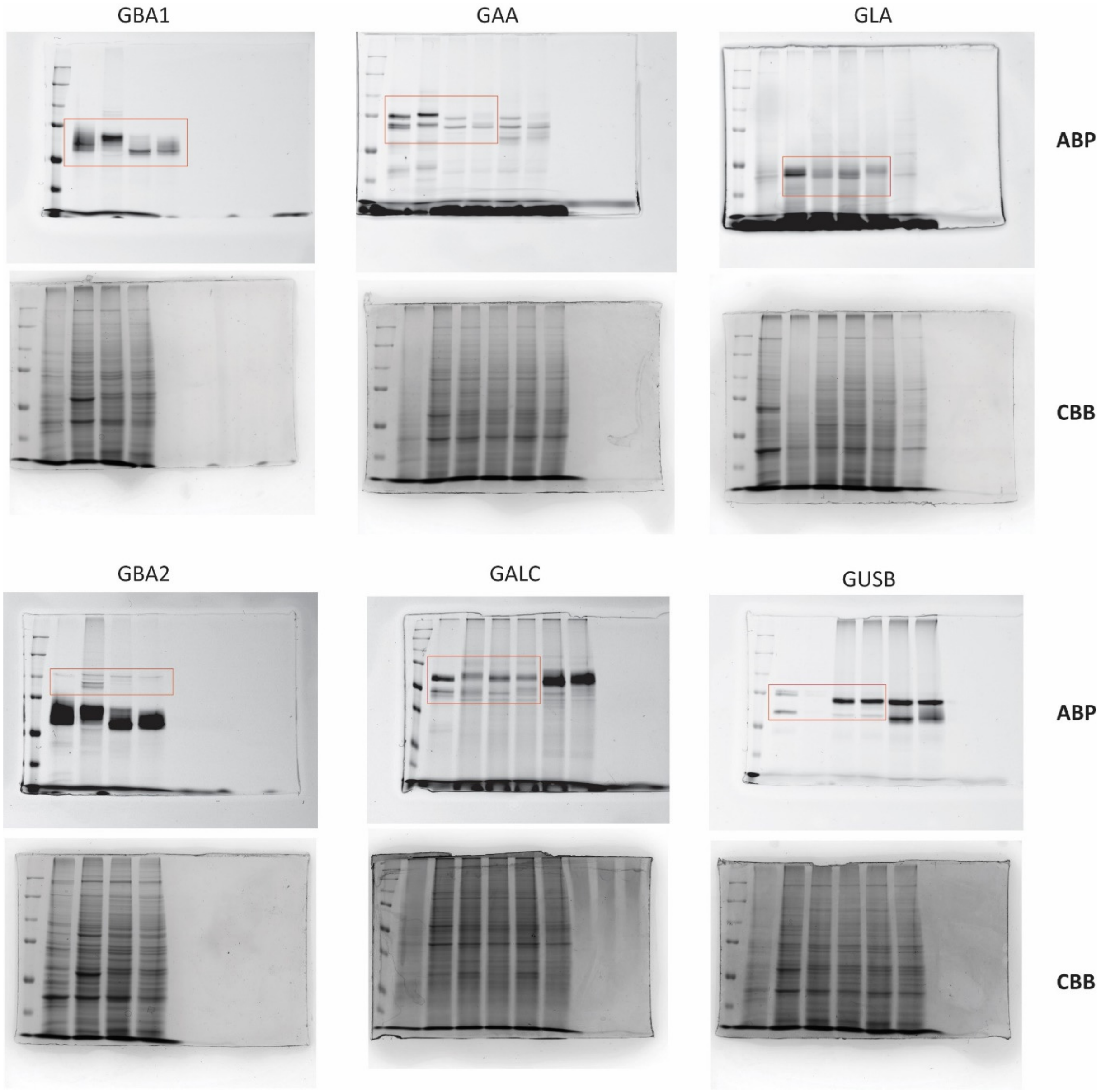

Figure S3. ABP labelling of lysosomal glycosidases in RAW264.7, J774A.1, THP-1 and human monocyte-derived macrophage lysates. Full gel with fluorescence detected ABP-labelled glycosidases GBA1 (ABP **5**), GAA (ABP **9**), GLA (ABP **8**), GBA2 (ABP **6**), GALC (ABP **7**) and GUSB (ABP **10**) with their corresponding Coomassie (CBB) staining of proteins. The red box indicates part of the gel which is shown in figure 2 (main text). Gels representative example of n = 3 experiments.

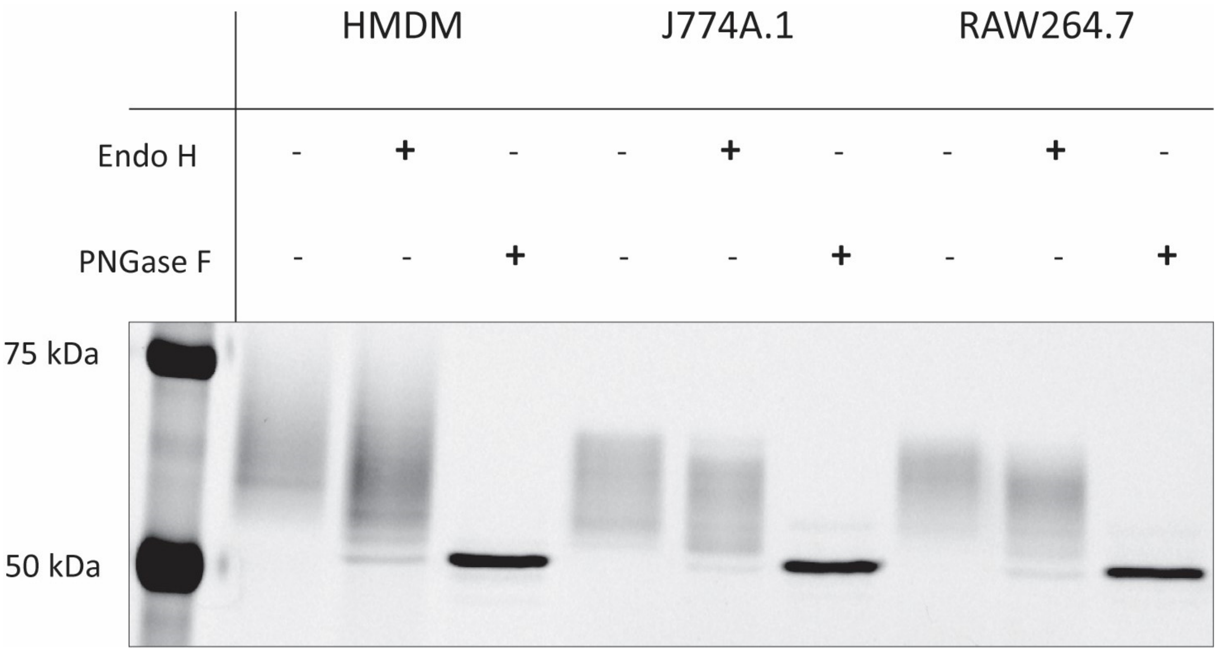

Figure S4. Activity based probe labelling of β-glucocerebrosidase after glycan removal. In gel fluorescence of β-glucocerebrosidase labelled with an activity based probe in lysates of human monocyte-derived macrophages (HMDM), J774A.1 and RAW264.7 cells. After labelling the lysates for 60 min at 37 °C, the samples were either treated with EndoH or PNGaseF for the removal of n-linked glycans. Gel representative example for n = 2 experiments.

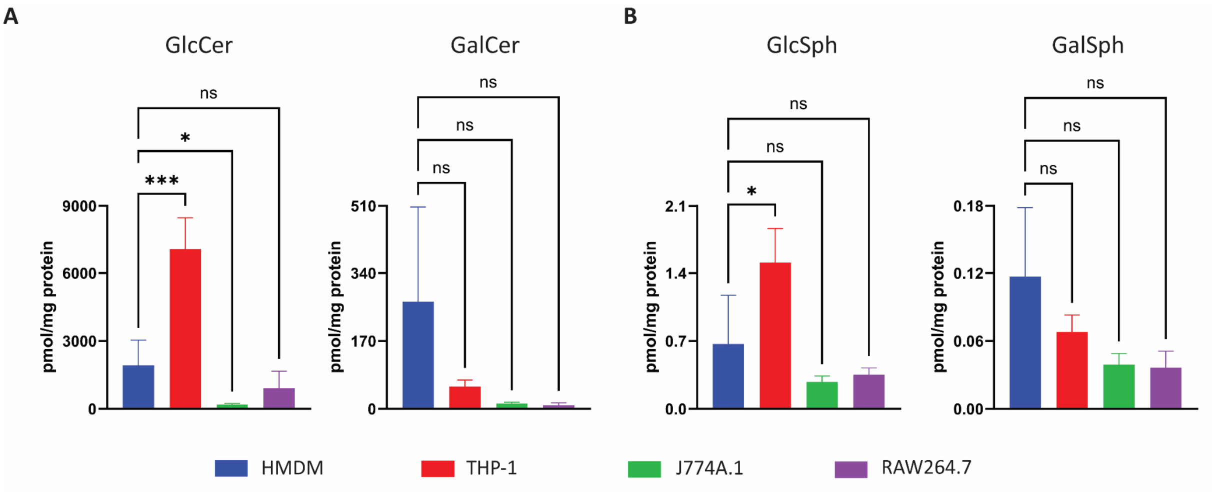

Figure S5. Different glycosphingolipids measured in various macrophages. Lysates of human monocyte-derived macrophages (HMDM), THP-1 macrophages, J774A.1 and RAW264.7 cells were measured to obtain difference between glucose and galactose glycosphingolipids. A). Measurement of glucosylceramide (GlcCer) and galactosylceramide (GalCer) and B). corresponding lyso-lipids glucosylsphingosine and galactosylsphingosine. Values are expressed in pmol/mg protein as mean ± SD with n = 3 and technical duplicates. Statistics were performed if samples were above the limit of detection with significance: ns = non significant, $^*P < 0.033$, $^{**}P < 0.002$ and $^{***}P < 0.001$.

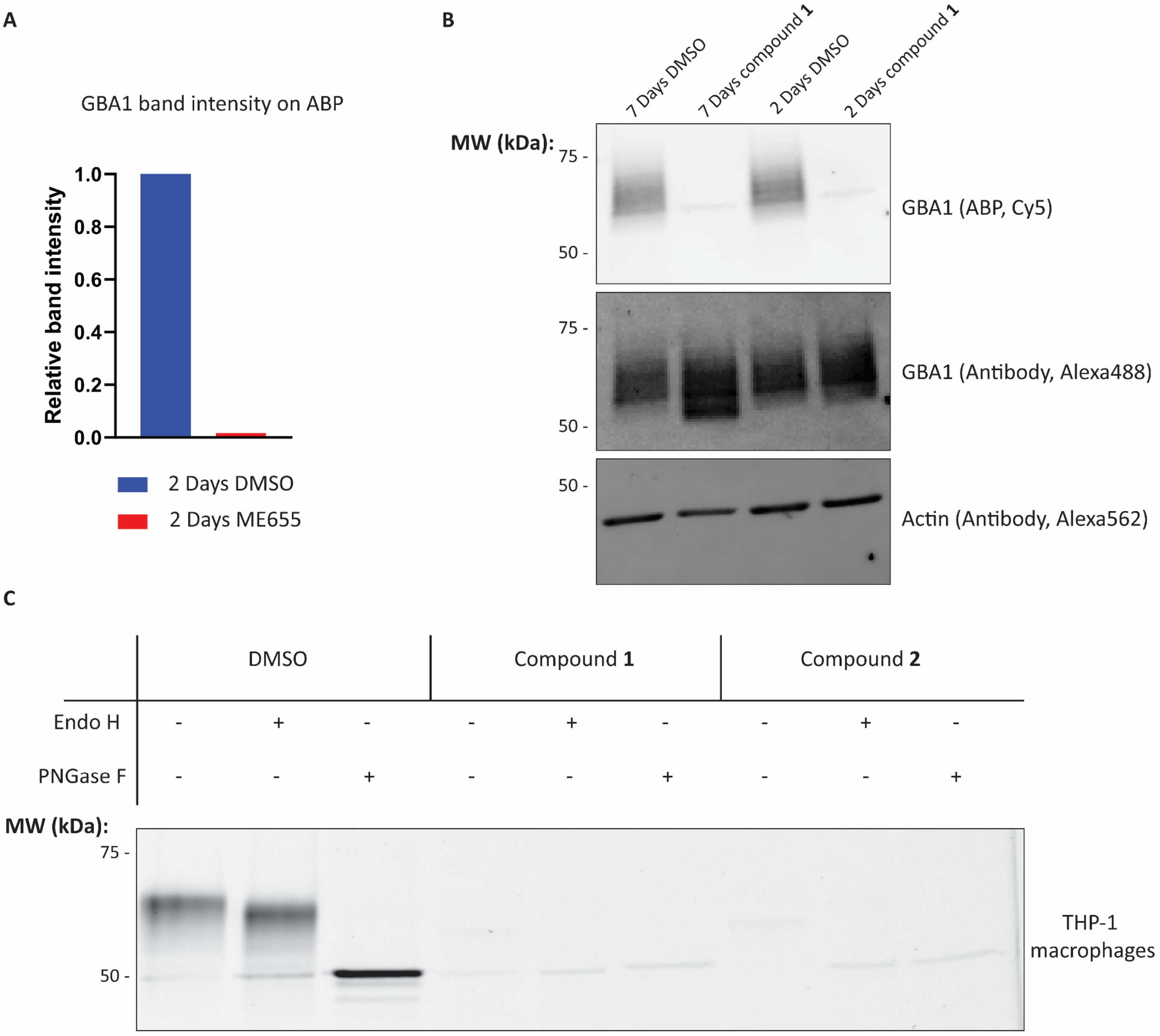

Figure S6. Gaucher model generation by specific inhibition of glucocerebrosidase. PMA-differentiated THP-1 macrophages were incubated for 48h with compound **1** *In situ* to inhibit glucocerebrosidase (GBA1). A). Relative intensity of GBA1 activity based probe (ABP) from main figure 5A normalized towards β-actin. B). Treatment with ABP **5** shows inhibition of GBA1 while antibody staining confirms equal GBA1 in each sample. C). THP-1 macrophages treated with compound **1** or **2** and followed by (partial) deglycosylation of GBA1 using EndoH or PNGase F treatment. Gels representative example of n = 3 experiments for A,B and n = 1 for C.

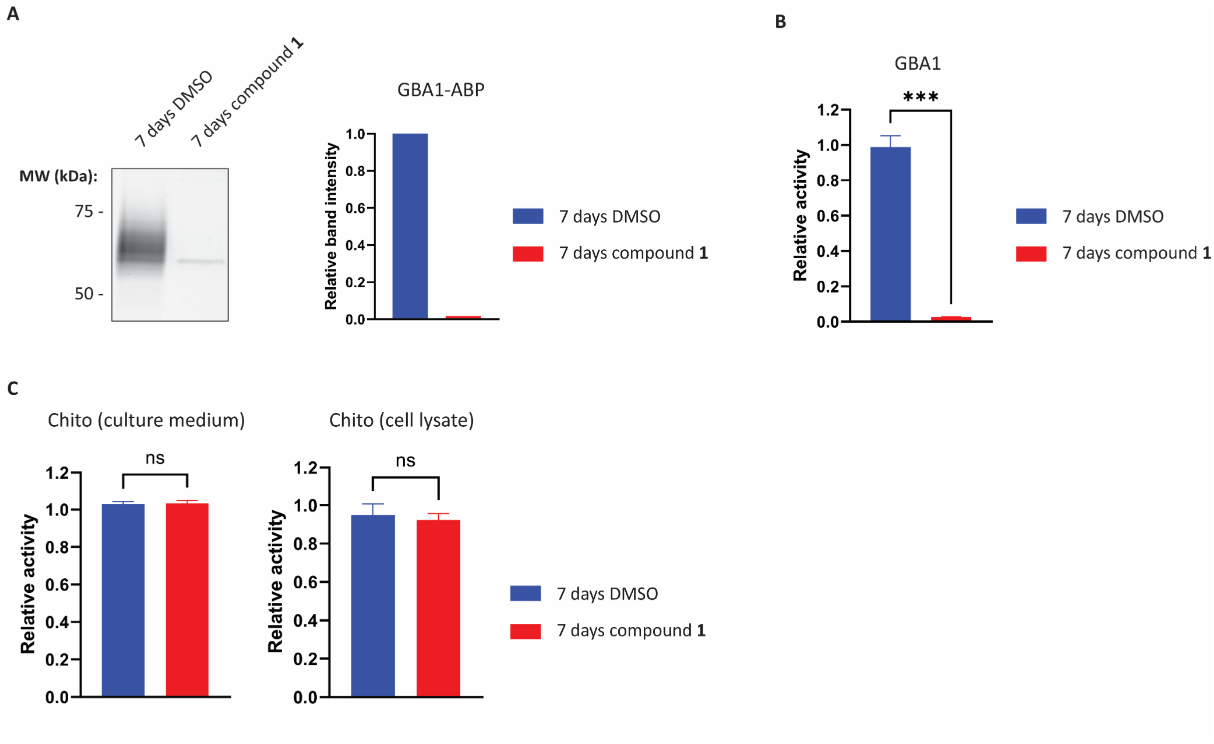

Figure S7. Analysis of human monocyte derived macrophages (HMDMs) cultured with 100 nM GBA1 inhibitor **1** for 7 days. HMDMs were harvested at 21 days old and lysates were analysed A). Unbound GBA1 detected with ABP **5** and relative GBA1 band intensity normalised on β-Actin western blot shown in figure S5B. B). residual relative GBA1 enzyme activity. C). Relative chitotriosidase (Chito) enzyme activity in cell lysate and culture medium. Gel is representative example of n = 2. Values are represented as mean ± SD with n = 2 and technical triplicates. For statistical analysis a two-tailed unpaired student's t-test was performed with statistical significance of: ns = non significant, *P < 0.033, **P < 0.002 and *** P < 0.001.

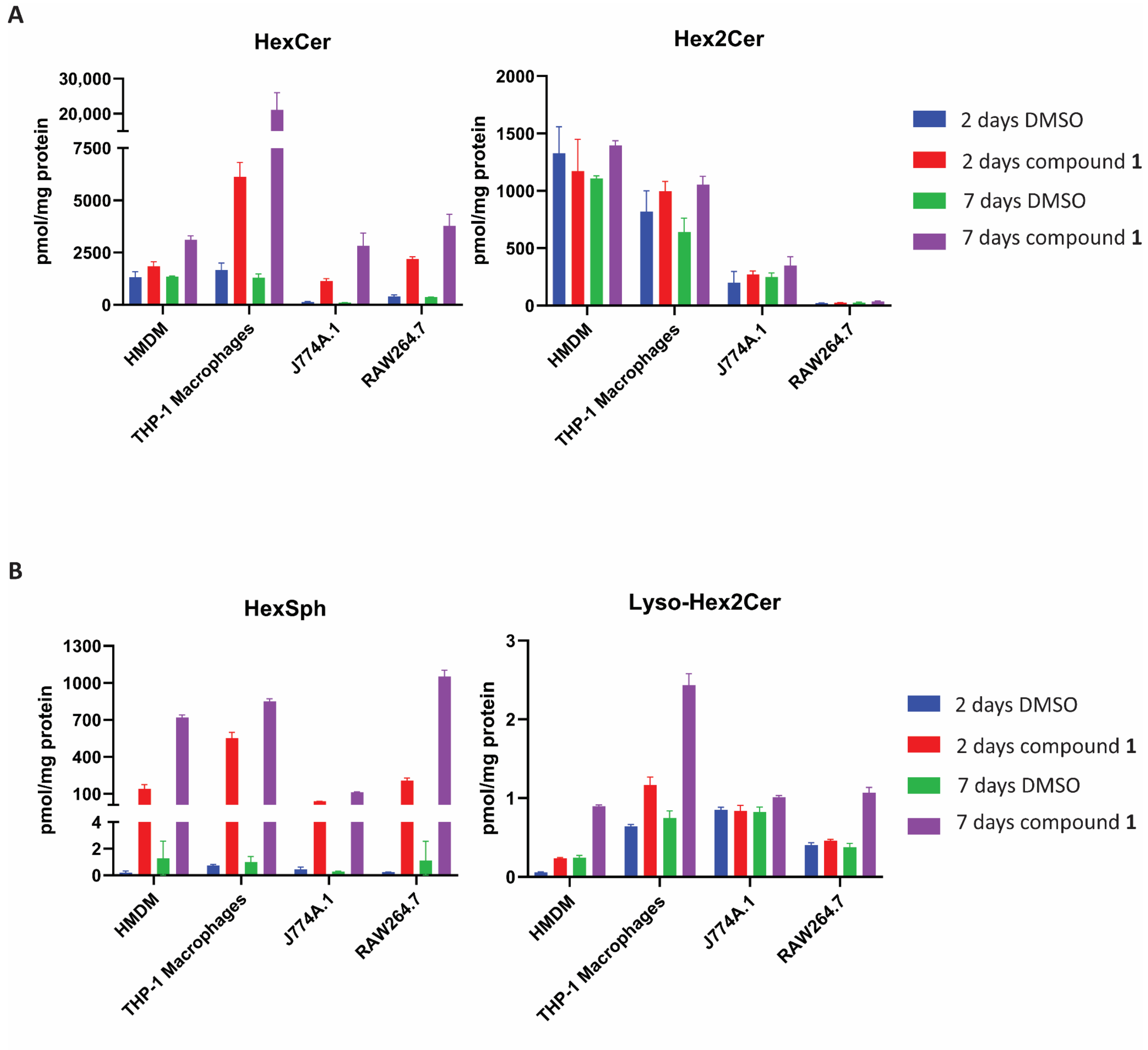

Figure S8. Sphingolipids and lyso-sphingolipids levels (pmol/mg protein) in macrophage models after inhibition of glucocerebrosidase. Human monocyte-derived macrophages (HMDM), THP-1 macrophages, RAW264.7 and J774A.1 cells were treated with compound **1** for 2 days or 7 days. Afterwards a selection of A). Sphingolipids and B). corresponding lyso-sphingolipids were measured. Values are expressed as mean ± SD with n = 3.

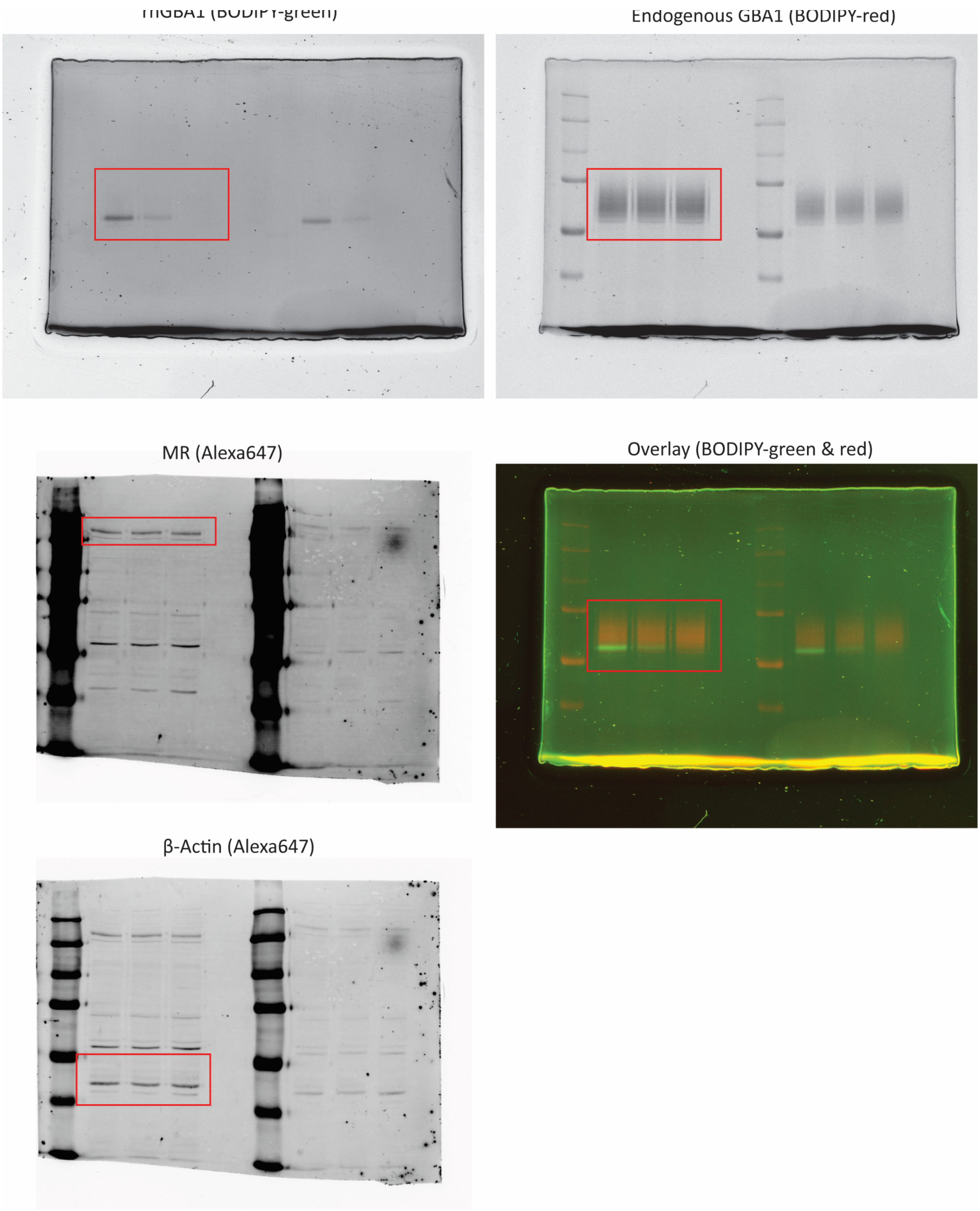

Figure S9. ABP and western blot of therapeutic human glucocerebrosidase feeding to human monocyte-derived macrophages. Full gel with fluorescence detected ABP-labelled endogenous glucocerebrosidase ABP **4** (red) and internalized therapeutic human glucocerebrosidase ABP **3** (green). And corresponding full western blot of the ABP-gels stained for mannose-receptor (MR) and β-Actin. The red box indicates the part of the gel shown in figure 6 (main text). Gels representative example of n = 3.
